# PRagMatic Pediatric Trial of Balanced vs nOrmaL Saline FlUid in Sepsis: study protocol for the PRoMPT BOLUS randomized interventional trial

**DOI:** 10.1186/s13063-021-05717-4

**Published:** 2021-11-06

**Authors:** Scott L. Weiss, Fran Balamuth, Elliot Long, Graham C. Thompson, Katie L. Hayes, Hannah Katcoff, Marlena Cook, Elena Tsemberis, Christopher P. Hickey, Amanda Williams, Sarah Williamson-Urquhart, Meredith L. Borland, Stuart R. Dalziel, Ben Gelbart, Stephen B. Freedman, Franz E. Babl, Jing Huang, Nathan Kuppermann

**Affiliations:** 1 Department of Anesthesiology and Critical Care, The Children’s Hospital of Philadelphia, Perelman School of Medicine at the University of Pennsylvania, Philadelphia, PA USA; 2 The Children’s Hospital of Philadelphia Pediatric Sepsis Program, Philadelphia, PA USA; 3 Department of Pediatrics, The Children’s Hospital of Philadelphia, Perelman School of Medicine at the University of Pennsylvania, Philadelphia, PA USA; 4 Department of Emergency Medicine, The Royal Children’s Hospital, Parkville, Victoria Australia; 5 Departments of Pediatrics and Critical Care, The University of Melbourne, Parkville, Victoria Australia; 6 Murdoch Children’s Research Institute, Melbourne, Victoria Australia; 7 Departments of Pediatrics and Emergency Medicine, Alberta Children’s Hospital Research Institute, Cumming School of Medicine, University of Calgary, Calgary, AB Canada; 8 Department of Biomedical and Health Informatics, Data Science and Biostatistics Unit, The Children’s Hospital of Philadelphia, Philadelphia, PA USA; 9 Divisions of Emergency Medicine and Pediatrics, Perth Children’s Hospital, School of Medicine at the University of Western Australia, Crawley, Australia; 10 Departments of Surgery and Pediatrics: Child and Youth Health, Starship Children’s Hospital, University of Auckland, Auckland, New Zealand; 11 Australian and New Zealand Intensive Care Society Paediatric Study Group, Camberwell, Australia; 12 Paediatric Intensive Care Unit, Royal Children’s Hospital, Parkville, Victoria Australia; 13 Sections of Pediatric Emergency Medicine and Gastroenterology, Departments of Pediatrics and Emergency Medicine, Cumming School of Medicine, University of Calgary, Calgary, AB Canada; 14 Department of Biostatistics, Epidemiology, and Informatics, Perelman School of Medicine at the University of Pennsylvania, Philadelphia, PA USA; 15 Department of Emergency Medicine and Pediatrics, UC Davis School of medicine and UC Davis Health, Sacramento, CA USA

**Keywords:** Sepsis, Septic shock, Pediatric, Intravenous fluid, Crystalloid, Saline, Renal failure, Pragmatic trial

## Abstract

**Background/aims:**

Despite evidence that preferential use of balanced/buffered fluids may improve outcomes compared with chloride-rich 0.9% saline, saline remains the most commonly used fluid for children with septic shock. We aim to determine if resuscitation with balanced/buffered fluids as part of usual care will improve outcomes, in part through reduced kidney injury and without an increase in adverse effects, compared to 0.9% saline for children with septic shock.

**Methods:**

The Pragmatic Pediatric Trial of Balanced versus Normal Saline Fluid in Sepsis (PRoMPT BOLUS) study is an international, open-label pragmatic interventional trial being conducted at > 40 sites in the USA, Canada, and Australia/New Zealand starting on August 25, 2020, and continuing for 5 years. Children > 6 months to < 18 years treated for suspected septic shock with abnormal perfusion in an emergency department will be randomized to receive either balanced/buffered crystalloids (intervention) or 0.9% saline (control) for initial resuscitation and maintenance fluids for up to 48 h. Eligible patients are enrolled and randomized using serially numbered, opaque envelopes concurrent with clinical care. Given the life-threatening nature of septic shock and narrow therapeutic window to start fluid resuscitation, patients may be enrolled under “exception from informed consent” in the USA or “deferred consent” in Canada and Australia/New Zealand. Other than fluid type, all decisions about timing, volume, and rate of fluid administration remain at the discretion of the treating clinicians. For pragmatic reasons, clinicians will not be blinded to study fluid type. Anticipated enrollment is 8800 patients. The primary outcome will be major adverse kidney events within 30 days (MAKE30), a composite of death, renal replacement therapy, and persistent kidney dysfunction. Additional effectiveness, safety, and biologic outcomes will also be analyzed.

**Discussion:**

PRoMPT BOLUS will provide high-quality evidence for the comparative effectiveness of buffered/balanced crystalloids versus 0.9% saline for the initial fluid management of children with suspected septic shock in emergency settings.

**Trial registration:**

PRoMPT BOLUS was first registered at ClinicalTrials.gov (NCT04102371) on September 25, 2019. Enrollment started on August 25, 2020.

**Supplementary Information:**

The online version contains supplementary material available at 10.1186/s13063-021-05717-4.

## Background

Crystalloid fluid is part of the initial resuscitation for 25 million children with septic shock worldwide each year [1]. Options for crystalloid resuscitation include 0.9% saline or balanced/buffered fluids (lactated Ringer’s [LR], Hartmann’s solution, PlasmaLyte). Saline contains a supra-physiologic concentration of chloride and low strong ion difference (SID) that induce hyperchloremic metabolic acidosis, decrease renal blood flow, and promote inflammation [2, 3]. In contrast, balanced/buffered fluids have less chloride, some additional electrolytes, and a higher SID due to an anion buffer. Balanced/buffered fluids have been associated with better outcomes compared to 0.9% saline, including decreased acute kidney injury (AKI), need for renal replacement therapies (RRT), and death in adult [4–10] and pediatric [11] studies, while others reported no benefit [12] or harm [13]. Notably, 0.9% saline remains the most commonly used fluid for children with septic shock due to historical preference, universal availability, ease of use, drug compatibility, lower cost, and lack of definite benefit for alternatives [14–16].

The Isotonic Solutions and Major Adverse Renal Events Trial (SMART) found a small, but significant, reduction in major adverse kidney events within 30 days (MAKE30) with the use of balanced/buffered fluids compared to 0.9% saline in critically ill adults [17]. The largest benefits were in sepsis, with 4.2% lower mortality for balanced/buffered fluids. A parallel trial found similar benefits in non-critically ill adults [18]. In children, however, the two largest studies of fluid type in sepsis are observational and reached opposing conclusions [19, 20]. Consequently, recent guidelines issued a weak suggestion for balanced/buffered crystalloids while calling for studies to compare the effectiveness of different fluid types in pediatric septic shock [21, 22].

The primary aim of the Pragmatic Pediatric Trial of Balanced versus Normal Saline Fluid in Sepsis (PRoMPT BOLUS) is to determine if resuscitation with balanced/buffered fluids as part of usual care will improve outcomes compared to 0.9% saline for children with septic shock. Secondary aims are to compare the relative safety and determine the differential effect of each fluid type on kidney injury biomarkers in children with septic shock. We hypothesize that balanced/buffered fluid resuscitation will reduce MAKE30 (a composite of death, new RRT, or persistent kidney dysfunction) compared to 0.9% saline, in part through reduced kidney injury, without an increase in adverse effects. Notably, even a small clinical benefit would be important because sepsis is common, life-threatening, and the proposed practice change is inexpensive and readily available.

## Methods

This manuscript was written in accordance with Standard Protocol Items: Recommendations for Interventional Trials (SPIRIT) guidelines (Additional file 1) and the 2010 CONSORT recommendations for pragmatic trials [23].

### Trial design

**Fig. 1:**
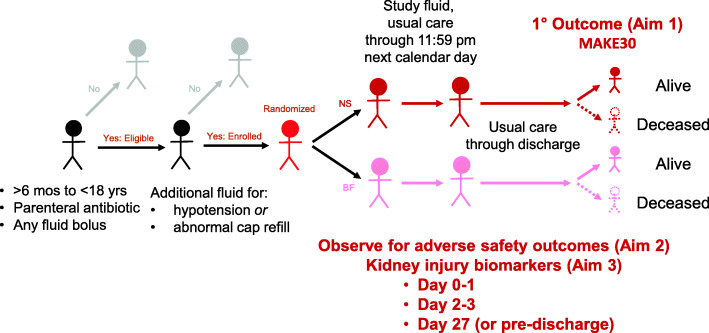
Schematic overview of the study design

**Fig. 2:**
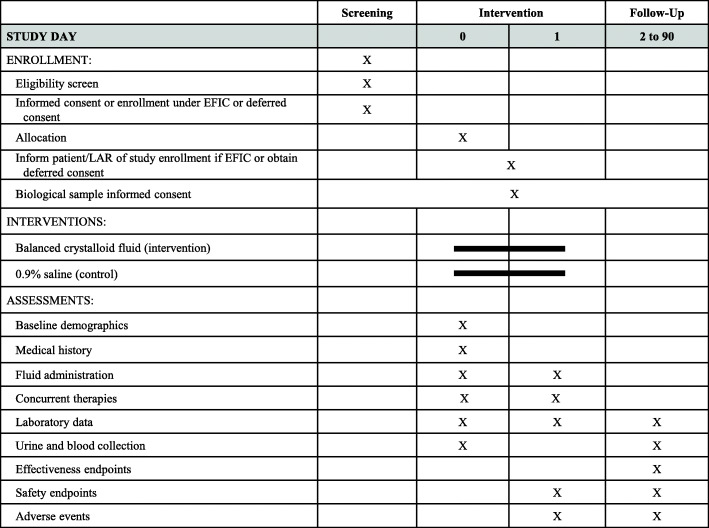
SPIRIT summary of study activities

PRoMPT BOLUS is a multicenter, open-label, pragmatic interventional trial in which children with suspected septic shock are randomized to receive either 0.9% saline or balanced/buffered crystalloid fluids for initial resuscitation and maintenance fluids in addition to usual care. A pragmatic design [24] was selected to optimize cost-efficiency and generalizability (Table 1), and in a pilot study, was demonstrated to be feasible to implement within a busy pediatric emergency department [25]. Figure 1 shows an overview of the study design and Fig. 2 is the SPIRIT summary of study activities.

**Table 1:** Key features of explanatory versus pragmatic clinical trials^a^

Study element	Explanatory clinical trial	Pragmatic clinical trial	Considerations for PRoMPT BOLUS
Objective	Mechanism or efficacy	Effectiveness	Comparative effectiveness of two existing standards of care
Study population	Restrictive, homogeneous (“ideal” target population)	Heterogeneous (“real-world” clinical practice)	Children treated with fluid therapy for suspected septic shock with abnormal perfusion as per treating clinician judgment
Intervention	Inflexible, strictly enforced	Usual practice #1	Balanced/buffered crystalloid fluid (site/clinician preference to use lactated Ringer’s, PlasmaLyte, or Hartmann’s)
Comparison	Inflexible, placebo, or usual practice	Usual practice #2	0.9% saline
Data collection	Extensive, labor-intensive	Targeted, limited	Targeted and limited to essential data to ensure balance in key covariates between study groups and to collect all outcomes
Protocol implementation and oversight	Dedicated study team assists with enrollment and study procedures	Treating clinicians carry out study procures embedded within clinical practice	Emergency physicians will be trained to screen, enroll, randomize, and initiate study fluids for bolus and maintenance therapy
Protocol adherence	Closely monitored with tight parameters	Unobtrusive or none	Guidance provided to treating clinicians to use randomized study fluid for bolus and maintenance fluid therapy from randomization through 11:59 PM of the following calendar day; non-study fluids allowed for specific clinical indications at clinicians’ judgment. Final adherence defined as receipt of ≥75% of total crystalloid administered by type assigned in the intervention phase.
Outcome(s)	Specialized experts often involved in quantifying study endpoints that are direct consequence of intervention	Endpoints are objective, clinically meaningful, and easily measured as part of routine clinical practice	MAKE30, mortality, hospital-free days, adverse events, and biomarkers all defined with objective criteria; effectiveness and safety outcomes all patient-centered.
Analysis	Intention-to treat with interpretation that intervention improves outcomes under “ideal” conditions	Intention-to-treat with interpretation that intervention improves outcomes under “usual” conditions	Intention-to-treat analysis will be interpreted as the comparative effectiveness between predominant—rather than exclusive—use of balanced/buffered fluids versus 0.9% saline
Generalizability	Variable (though often low)	Variable (though typically high)	Expected to be highly generalizable
Costs	Relatively high	Relatively low	Relatively low for large clinical trial

^a^Adapted from Thorpe et al *Journal of Clinical Epidemiology* 2009

### Study sites and timeline

This study is being conducted at > 40 sites from the Pediatric Emergency Research Networks, including the Pediatric Emergency Care Applied Research Network (PECARN) in the United States (US), Pediatric Emergency Research Canada (PERC), and Pediatric Research in Emergency Departments International Collaborative (PREDICT) in Australia/New Zealand. An international steering committee comprised of the overall study principal investigators (SLW, FB) and network-specific principal investigators from each geographic network will oversee all study activities. Enrollment started August 25, 2020, and is anticipated to continue for 5 years. Patients will only be enrolled in an emergency department (ED), where initial resuscitation of suspected septic shock will begin. Ongoing inpatient management of septic shock may occur on any hospital ward or intensive/critical care unit.

### Participants

All patients > 6 months to < 18 years treated for suspected septic shock with parenteral antibiotics and at least one fluid bolus for abnormal perfusion in a study site ED will be eligible for study enrollment. The lower age was selected to adhere to the US Food and Drug Administration (FDA) warning that infants < 6 months may have reduced hepatic capacity to metabolize exogenous lactate in LR. “Suspected septic shock” is operationalized as a) the treating clinician’s diagnosis of septic shock and/or treatment that includes parenteral antibiotic and fluid resuscitation for abnormal perfusion or b) a sepsis alert on a site-specific screening tool with clinician confirmation to proceed with treatment for suspected septic shock. Abnormal perfusion is defined as the treating clinician’s judgment that hypotension or abnormal (either “flash” or “prolonged”) capillary refill is present. To reflect usual clinical practice, we do not define thresholds for hypotension or abnormal capillary refill but rather defer to the treating clinician’s discretion to differentiate abnormal from normal. Recognizing that fluid resuscitation may start prior to ED arrival, only those for whom total volume of crystalloid fluid administration can be confirmed as ≤40 mL/kg will be eligible.

Exclusion criteria are (1) clinician judgment that the patient’s condition deems it unsafe to administer either 0.9% saline or balanced/buffered fluids (see Additional file 2), (2) known pregnancy at time of assessment for eligibility, (3) known prisoner at time of assessment for eligibility (in USA, Australia/New Zealand), (4) patient is a ward of the state (in Australia/New Zealand), (5) known allergy to either fluid type, or (6) prior indication that patient would not agree to be enrolled in the study.

### Research ethics approval

A single Institutional Review Board at The Children’s Hospital of Philadelphia (CHOP) will provide regulatory approval and oversight for all US study sites. In Canada, the University of Calgary will sponsor regulatory approval through Health Canada, while each site’s Research Ethics Board will oversee local human subjects’ protections (except sites in Ontario, for which centralized approval through Clinical Trials Ontario will be obtained). In Australia and New Zealand, a single ethics committee will provide regulatory oversight within each jurisdiction. Protocol modifications approved by the international steering committee will be first reviewed by the CHOP Institutional Review Board and, following approval, will be distributed by the network-specific principal investigators to all other regulatory bodies in the USA (local site Institutional Review Boards, FDA), Canada, Australia, and New Zealand, as well as to local investigators.

### Consent

Prospective written informed consent will be sought from a legally authorized representative (LAR, e.g., at least one parent) prior to enrollment *when sufficient time permits*. However, because of the life-threatening nature of septic shock and narrow therapeutic window to commence fluid resuscitation, enrollment may proceed in the USA under the federal “Exception From Informed Consent” (EFIC, 21 CFR 50.24) for emergency research [26] and in Canada and Australia/New Zealand with “deferred consent” in compliance with the Canadian Tri-Council Policy Statement-2 [27] and National Statement on Ethical Conduct in Human Research [28], respectively. Such enrollment methods are ethically suitable for emergent, life-threatening conditions when patients may benefit from the research, available treatments are unproven/unsatisfactory, and obtaining prospective informed consent is not feasible [25, 29–32]. A waiver of assent will be requested due to the critical nature of illness in septic shock.

To comply with federal and ethical guidelines, community consultation and public disclosure were conducted in the USA (available at https://www.regulations.gov/docket/FDA-1995-S-0036) and community consultation was conducted in Australia/New Zealand to obtain input from and inform the local community prior to beginning enrollment [33, 34]. All EFIC studies are also subject to additional regulation through the US FDA, which approved the study protocol under an Investigational New Drug (IND #136978) application.

Participants enrolled under EFIC (USA) or deferred consent (Canada, Australia/New Zealand) will be notified of enrollment and study procedures as soon as feasible and offered the right to withdraw from or continue in the study. For study participants who die prior to the post-enrollment discussion in the USA or deferred consent in Canada or Australia/New Zealand, all reasonable attempts will be made to inform the LAR after the participant’s death, unless a waiver has been granted in local jurisdictions. However, successful LAR contact will not be required to include data in the analysis. At a subset of sites collecting biological samples, separate written informed consent will be obtained for the measurement of blood and urine biomarkers.

### Randomization and allocation concealment

Patients will be allocated in equal numbers to groups by a permutated-block randomization sequence using blocks of 2, 4, or 6, stratified by site and generated by the lead trial statistician (JH). Block lengths of 2, 4, or 6 will be selected randomly with equal probability, and the treatment sequence within blocks will be randomly shuffled until a list of 300–600 is generated for each site. For large sites that expect enrollment from separate areas of the emergency department, separate randomization lists will be generated for each recruitment area. Study group allocation will be concealed using serially numbered, opaque envelopes, which provides an efficient process for quickly randomizing participants concurrent with ongoing clinical management. Treatment allocation to 0.9% saline or balanced/buffered fluids will be revealed after eligibility for enrollment has been confirmed.

### Intervention

The intervention to be tested is predominant fluid resuscitation with balanced/buffered fluids (LR, Hartmann’s, or PlasmaLyte, as per local site preference) compared to the “usual practice” of predominant 0.9% saline resuscitation. Other than prescribing which crystalloid fluid type to use, the intervention will be implemented without efforts to standardize timing and volume of fluid resuscitation or other components of clinical care. Thus, all decisions to administer fluid, as well as timing, volume, and rate of fluid administration, remain at the discretion of the treating clinicians. Hospital fluid supplies will be used for this study without any change to packaging or labeling. Participants will be randomized to receive either balanced/buffered fluids or 0.9% saline for all bolus and maintenance fluid administration starting from randomization through 11:59 pm on the following calendar day. Maintenance fluids are included because these constitute a substantial proportion of total crystalloid fluid administration [35], including 40% of total crystalloids as maintenance fluids in our pilot study [25]. If the treating clinician deems it unsafe to continue study fluid or identifies a clinical indication that requires specific fluid or electrolyte therapy (e.g., hyponatremia, hyperkalemia, hypercalcemia, cerebral edema), non-study fluids are permitted. Use of non-isotonic fluids (e.g., 0.45% saline) is not recommended as standard maintenance fluids in children [36] and will be discouraged during the intervention phase. Colloid fluids and blood products may be administered at the discretion of the treating team.

Ending the intervention phase at 11:59 PM on the calendar day following randomization ensures all participants are eligible to receive study fluid for a minimum of 24 and a maximum of 48 h. This represents the time-window from the presentation during which most fluid resuscitation is completed for septic shock [17] and provides a pragmatic endpoint that signals the end of the intervention phase.

### Adherence

All fluid administered through the end of the intervention window will be recorded. Adherence to the intervention is defined as receipt of ≥75% of total crystalloid fluid as the randomized fluid type during the interventional window. For example, participants randomized to balanced/buffered fluids are considered adherent to the intervention if they receive ≥75% of their total crystalloid as balanced/buffered fluids starting from the time of randomization through 11:59 PM on the following calendar day. Locally, all sites will establish a work-flow to communicate study enrollment and promote adherence across providers and hospital locations. Such strategies include verbal and/or written hand-offs between providers, use of a templated orderset indicating PRoMPT BOLUS study enrollment and treatment allocation, and automated electronic reminders to clinicians at the time an order is placed for non-study crystalloid fluids during the intervention window. Each participant’s adherence to the intervention will be monitored centrally, and the mean adherence to the intervention for all participants within each site will be regularly reported back to local investigators. Targeted education will be undertaken if < 80% of participants in either study arm meet the adherence criterion. If < 70% of participants meet the adherence criterion over two consecutive months, the site will be placed on monitored probation with additional efforts undertaken to understand and correct challenges to adherence. Sites that continue to fail to meet the adherence threshold in at least 80% of enrolled participants may be removed from study participation.

### Blinding

Study participants, clinicians, and investigators will not be blinded to treatment allocation. Blinding study fluid is not pragmatic [17, 18] and unlikely to even be possible because available laboratory values overtly reflect the crystalloid being used [9]. However, randomization of individuals following study enrollment will minimize selection bias related to pre-enrollment awareness of study group assignment. The lead biostatistician (JH), however, will remain blinded to group assignment for purposes of data analysis.

### Data collection and management

Data will be extracted from the medical record and recorded onto a standardized case report form. Each network will oversee data collection and quality from its respective sites. In the USA, data will be collected through FDA-compliant Advarra Electronic Data Capture (Columbia, MD) hosted at CHOP. In Canada and Australia/New Zealand, data will be collected in Research Electronic Data Capture (REDCap, Vanderbilt University; Nashville, TN) hosted at the University of Alberta and the Murdoch Children’s Research Institute, respectively [37]. However, all sites will collect the same data elements using a common data dictionary. All study data will be password-protected and coded with a study number to ensure confidentiality. Periodic verification of study data against source documents will be undertaken within each network to certify data accuracy. The US Data Coordinating Center (DCC) will centrally manage all data exported from Advarra and REDCap to ensure harmonization for analyses. Consistent with the tenets of a pragmatic trial, data collection will be brief, targeting key patient characteristics, fluid administration, and outcomes (Additional file 3).

### Outcomes

The primary outcome will be MAKE30—a composite of death from any cause, initiation of RRT, or persistent kidney dysfunction—at either 30 days following study enrollment or hospital discharge, whichever comes first (Table 2) [38]. RRT will include treatment (or attempt to treat if the participant does not tolerate treatment) with any renal replacement modality during the hospitalization that was not a continuation of pre-hospital chronic therapy, censored at 30 days after randomization. Persistent kidney dysfunction will be measured as final creatinine ≥200% of baseline (i.e., at least a doubling of baseline creatinine) *and* a minimum absolute increase of ≥0.3 mg/dL. Baseline serum creatinine will be recorded as the lowest creatinine available between 12 months and 24 h prior to enrollment or, for participants without such data, an imputed value using established median values for age and sex [39]. Secondary efficacy, safety, and biologic outcomes are listed in Table 2.

**Table 2:** Study outcomes

Outcome	Definition
Primary outcome	
MAKE30 (primary outcome)	Major adverse kidney events at 30 days (defined as at least one of the following): • Death • New renal replacement therapy • Persistent kidney dysfunction at hospital discharge or 30 days (serum creatinine ≥2x baseline or median value for age if no baseline available *and* a minimum absolute increase of ≥0.3 mg/dL)
Secondary effectiveness outcomes	
Death	All-cause mortality at hospital discharge, 28 days, and 90 days^b^
Hospital length of stay	Days from hospital admission until discharge, censored at 90 days
Hospital-free days out of 28 days	Days between enrollment and day 28 in which patient was alive and out of the hospital
New inpatient renal replacement therapy	New continuous renal replacement therapy, hemodialysis, or peritoneal dialysis
Persistent kidney dysfunction at hospital discharge	Serum creatinine ≥2x baseline or median value for age if no baseline^a^ available, censored at 30 days
Secondary safety outcomes^c^	
Hyperlactatemia	> 4 mMol/L
Hyperkalemia	> 6 mEq/L
Hypercalcemia	Ionized calcium > 1.35 mmol/L or total serum calcium > 12 mg/dL
Hypernatremia	> 155 mEq/L
Hyponatremia	< 128 mEq/L
Hyperchloremia	> 110 mEq/L
Thrombosis	Therapy for new arterial or venous thrombus with systemic anticoagulant OR Clotting of intravenous catheter in subjects receiving ceftriaxone and lactated Ringer’s
Cerebral edema	Therapy with hyperosmolar therapy (hypertonic saline and/or mannitol) for radiographic and clinical determination of new impending or present brain herniation
Secondary biologic outcomes^d^	
Urine neutrophil gelatinase-associated lipocalin	Biomarkers will be measured on the day of randomization (day 0), day 2, and day 27 (or prior to death or anticipated discharge, whichever comes first). Analyses will include between-group differences in absolute biomarker values at each timepoint and, to account for potential baseline differences, the percent change in biomarkers relative to measurements on study 0.
Urine kidney injury molecular-1	
Urine liver-type fatty acid-binding protein	
Urine interleukin-18	
Plasma cystatin C	

^a^Baseline creatinine will be determined for each study participant as the lowest recorded creatinine available between 12 months and 24 h prior to the index admission. For participants without such data available, an estimated baseline creatinine will be imputed using previously established median values for age and sex

^b^Mortality at 90 days will be measured using vital status obtained from the medical record at all sites, as well as the US National Death Index and Canadian provincial vital statistics if vital status cannot be confirmed using medical records alone

^c^Safety outcomes must occur within 4 calendar days of randomization, except thrombosis which must occur within 7 days of randomization

^d^Plasma and urine biomarkers of kidney injury will also be collected from study participants enrolled at a subset of sites

The rationale for MAKE30 as the primary endpoint is that mediation of kidney injury provides the major mechanistic pathway through which balanced/buffered fluids are likely to improve patient outcomes. In addition, the only prior comparable randomized clinical trials demonstrated a reduction in MAKE30 with the use of balanced/buffered fluids in adult patients with sepsis [17, 18]. Finally, MAKE30 provides an objective and easily measured outcome endorsed as a patient-centered endpoint for clinical trials [40].

### Adverse events

Adverse events (AEs) will be queried from the medical record at the end of the intervention phase and between study days 5–7. We will limit the time period for the determination of AEs because we are unaware of any biologically plausible reason to anticipate that either fluid type should lead to AEs remote from the immediate intervention period. AEs that are unexpected, serious, and at least possibly related to study fluid type will be reviewed by the international steering committee and reported promptly to regulatory bodies.

### Sample size

A baseline incidence of MAKE30 in children with septic shock predominantly resuscitated with 0.9% saline was determined based on a prior pediatric study (9.6% MAKE30) [39], the proportion of patients aged 18–21 years enrolled in the SMART trial (9.7% MAKE30) [17], and institutional registry data from two US study sites (5.0% and 7.8% MAKE30). We anticipate a conservative MAKE30 incidence of 6% among children treated in an ED for suspected septic shock largely resuscitated with 0.9% saline. Enrollment of 8800 total participants will provide 95% power to detect an absolute risk reduction (ARR) in MAKE30 from 6.0% for children treated with 0.9% saline to 4.3% for balanced/buffered fluids with a standard type I error of 0.05. This ARR corresponds to the 28% relative risk reduction in MAKE30 observed in the youngest subset of patients (18–21 years) enrolled in SMART. Due to the small anticipated ARR with potential for type II error, we purposely selected a high power of 95% for the primary endpoint. This sample size will also provide 84% power to detect a decrease in mortality from 3% with saline to 2% with balanced/buffered fluids and > 99% power to detect a 1-day reduction in hospital-free days.

Recruitment of this large sample size requires collaboration across > 40 sites from three research networks. Based on the enrollment of 85% of eligible patients in our pilot study [25], sites are anticipated to enroll > 80% of eligible patients. Recruitment will be monitored, with targeted support provided to sites that consistently fall below this target. Targeted support will include local investigators meeting with the lead investigators from their respective networks to review the process for screening, determination of eligibility criteria, education of local emergency clinicians who assess for eligibility, location of enrollment materials relative to patient care in the emergency department, and tracking of reasons for missed enrollments. If enrollment fails to demonstrate improvement toward or above the > 80% target despite these efforts, the site will be considered for removal from study participation and a replacement site will be identified.

### Data/safety monitoring and interim analysis

The study will be monitored by a Data Safety Monitoring Board (DSMB) comprised of 10 international members. Interim monitoring for the superiority of one treatment approach will be performed after enrollment of 15%, 40%, and 70% of total study participants. The significance levels at each interim analysis for the difference in MAKE30 between groups are 0.000000014, 0.00079, and 0.014, respectively, using the symmetric two-sided O’Brien-Fleming boundaries. The DSMB may also consider early termination of the trial if there is evidence of futility using an informal, conditional power approach [41].

### Statistical analysis

The primary outcome of MAKE30 will be reported using the Mantel-Haenszel (MH) relative risk, stratified by the study site. The primary analysis will be performed using the intention-to-treat (ITT) principle, including all randomized participants for whom the three components of the MAKE30 outcome are not missing, regardless of whether study fluid was administered. To account for the three planned interim analyses, the final analysis will use a significance level of 0.044 for the primary outcome of MAKE30.

Secondary outcomes will also be analyzed using the ITT approach incorporating methods of Benjamini and Hochberg to control for multiple analyses [42, 43]. The analysis plan for secondary outcomes is described in Additional file 4.

A per-protocol analysis will include all participants who receive at least 75% of total crystalloid fluid volume (bolus and maintenance) during the intervention window as the fluid to which the participant was randomized. Because the study intervention (fluid type) is available outside of the study, participants who continue to receive the same fluid to which they were randomized after withdrawal from the study protocol will remain eligible for the per-protocol analysis if they meet the ≥75% threshold. Participants who are found to have been ineligible for enrollment (e.g., age incorrect at initial eligibility assessment) or who receive no crystalloid fluid after randomization will be excluded from the per-protocol analysis. To account for potential confounding introduced into the per-protocol analysis, we will use generalized mixed effect regression models to adjust for known covariate imbalances. We will also conduct an analysis using instrumental variables to adjust for potential bias created by treatment contamination [44].

### Subgroup analyses

We will evaluate treatment differences among subgroups defined by the following stratification variables: age group, sex, cancer comorbidity, AKI at study enrollment, abnormal kidney function using day 0 biomarker measurements (Table 2), total fluid volume received during intervention window, and country of enrollment. If there is significant heterogeneity in treatment effect using the Breslow-Day test [45], we will report separate results within strata.

### Missing Data

The primary ITT analysis will use complete case data if the percentage missing the primary outcome is ≤5% of total enrollment *and* all three country-specific percentages missing the primary outcome is ≤7% of country-specific enrollment. If these criteria are not met, we will use multiple imputation for the primary analysis with data assumed to be missing at random unless available data contradict this assumption. If the primary outcome is missing from between 1% and 5% of total participants, we will perform an adjunctive analysis using multiple imputation in addition to the complete case analysis. In addition, we will examine differences between participants with and without missing data and conduct a sensitivity analysis using the inverse-probability-weighting (IPW) method to conduct multiple imputation for the missing data [46, 47]. New analyses performed in addition to those described above will be delimitated as *post hoc* and considered hypothesis-generating.

### Presentation of the results

Trial results will be communicated to the public through manuscript publication and ClinicalTrials.gov, including the full study protocol and statistical code. Authorship will adhere to the *International Committee of Medical Journal Editors* guidelines. To ensure widespread access to study findings, we will seek publication using “open access”. After the publication of main and secondary results, a public use dataset will be submitted to the *Eunice Kennedy Shriver* National Institute of Child Health and Development Data and Specimen Hub (DASH).

**Fig. 3:**
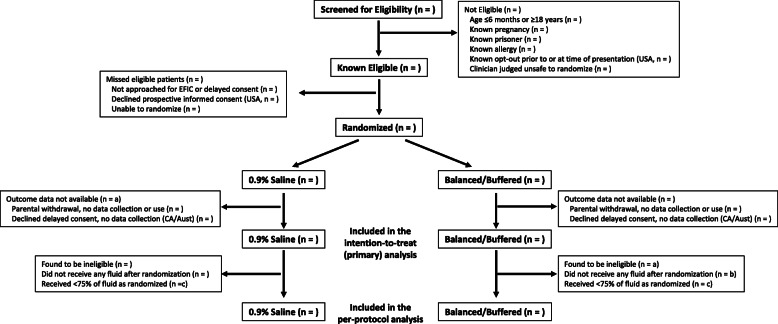
Flow diagram of patients progressing through the trial

The flow of participants through the study will be presented as in Fig. 3. Baseline characteristics will be presented by the treatment group, as in Table 3. Total, bolus, and maintenance volumes of crystalloid and other fluids and blood products will also be presented by the treatment group, as in Table 4. Laboratory data will be presented in figures displaying initial and follow-up values. All outcomes will be reported by the treatment group with both unadjusted frequencies and proportions, as well as with any statistical adjustments. Heterogeneity of treatment effect for subgroup analyses will be displayed within forest plots.

**Table 3:** Patient characteristics

Characteristic	0.9% saline	Balanced/buffered fluid
	(*n* = )	(*n* = )
Country of enrollment, *n* (%)		
USA		
Canada		
Australia/New Zealand		
Age in years, median (IQR)		
Age category, *n* (%)		
6 mos to < 1 year		
1 year to < 5 years		
5 years to < 12 years		
12 years to < 18 years		
Sex, *n* (%)		
Male		
Female		
Other/unknown		
Race, *n* (%)		
White		
Black		
Asian		
American Indian/Alaska Native		
Native Hawaiian/Other Pacific Islander		
Unknown/not reported^a^		
Ethnicity, *n* (%)		
Hispanic or Latino		
Aboriginal or Torres Strait Islander		
Maori		
Other^b^		
Unknown/not reported^a^		
Weight (kg), median (IQR)		
Comorbid conditions, *n* (%)		
Cancer (hematogenous or solid tumor)		
Bone marrow or solid organ transplant		
Cardiomyopathy or heart failure		
Pulmonary hypertension		
Kidney disease (not on dialysis)		
Neurologic dysfunction causing severe developmental delay		
Sickle cell disease		
Chronic ventilator dependence		
Indwelling central line		
Site of infection, *n* (%)		
Primary bloodstream		
Pneumonia or other lung infections		
Abdominal		
Genitourinary		
Central nervous system		
Skin/soft tissue		
Other infection		
Unknown site		
Alternative diagnosis (not infection)		
Positive blood culture (bacteremia) as either primary or additional site of infection, *n* (%)		
Concurrent Therapies (through study day 1, 11:59 pm)		
Antibiotics prior to study site ED arrival, *n* (%)		
Minutes to first antibiotic administration after ED arrival, median (IQR)^c^		
Ceftriaxone, *n* (%)		
Vasoactives, *n* (%)		
Corticosteroids, *n* (%)		
Bicarbonate or other buffers, *n* (%)		
Invasive mechanical ventilation^d^, *n* (%)		
Extracorporeal membrane oxygenation, n (%)		
Baseline creatinine (mg/dL)^e^, median (IQR)		
KDIGO acute kidney injury stage at enrollment, *n* (%)		
Stage 1		
Stage 2		
Stage 3		

*KDIGO*, Kidney Disease: Improving Global Outcomes

^a^Race and ethnicity not available from participants enrolled in Canadian sites

^b^“Other” ethnicity includes participants who identified as not Hispanic or Latino in the USA and not Aboriginal or Torres Strait Islander or Maori in Australia/New Zealand

^c^Includes only the subset of participants (*n* = ) who did not receive antibiotics prior to study site ED arrival

^d^Includes only invasive mechanical ventilation delivered through an endotracheal tube (oral or nasal), tracheostomy, laryngeal mask airway, or other invasive devices

^e^Baseline serum creatinine is the lowest creatinine available between 12 months and 24 h prior to study enrollment or, for participants without such data, an imputed value using established median values for age and sex

**Table 4:** Fluid administration

Characteristic	0.9% saline	Balanced/buffered fluid	Difference (95% CI)
	(*n* = )	(*n* = )	
Number of crystalloid fluid boluses, median (IQR)			
Total^a^			
Prior to randomization			
During intervention phase			
Crystalloid volume administered (mL/kg), median (IQR)			
Total fluid (bolus and maintenance)^a^			
Any crystalloid			
0.9% saline			
Balanced/buffered fluid			
Bolus fluid only			
Prior to randomization			
During intervention phase			
0.9% saline			
Balanced/buffered fluid			
Maintenance fluid only			
Any crystalloid			
0.9% saline			
Balanced/buffered fluid			
Other			
Colloid volume administered (mL/kg), median (IQR)			
Albumin 4%/5%			
Other			
Blood product volume administered (mL/kg), median (IQR)			
Red blood cells			
Platelets			
Plasma or cryoprecipitate			
Other			
Fluid volume categories^a^, *n* (%)			
Total crystalloid (bolus and maintenance)			
<60 mL/kg			
60–100 mL/kg			
>100 mL/kg			
Bolus fluid only			
<60 mL/kg			
60–100 mL/kg			
>100 mL/kg			
Protocol Adherence			
Received ≥75% of total crystalloid as randomized fluid type during intervention phase, *n* (%)			

^a^Includes fluid administered prior to randomization and during the intervention phase

## Discussion

PRoMPT BOLUS will provide high-quality evidence for the comparative effectiveness of buffered/balanced crystalloids versus 0.9% saline for the initial fluid management of children with suspected septic shock in emergency settings. Currently, there is insufficient evidence to support one crystalloid fluid type for pediatric shock resuscitation, which was acknowledged as a key knowledge gap by the 2020 Surviving Sepsis Campaign guidelines [21, 22]. Should balanced/buffered fluids improve outcomes compared to 0.9% saline, this practice change could be quickly implemented given that these fluids are readily accessible. Thus, the results of this trial could be rapidly incorporated into clinical practice with an immediate impact on the health of children worldwide. Conversely, because this trial is powered to detect small clinical differences, a finding of no difference between groups would signify that the initial choice of crystalloid fluid type is inconsequential for most children with septic shock during the initial 24-48 hour resuscitation period.

We selected a pragmatic design for several reasons [48, 49]. First, embedding enrollment into routine care will reduce cross-over by minimizing pre-enrollment exposure to non-study fluids. Second, a pragmatic protocol allows flexibility to execute the study across multiple sites, countries, and healthcare systems. Third, loosening reliance on a study team allows study procedures to continue across multiple hospital units and providers. Finally, generalizability is optimized by using a study protocol that mirrors the variance in clinical decision-making outside of this study.

The decision to use patient-level randomization rather than an alternative method based on systematic allocation (e.g., different fluid type in alternating months) or cluster randomization ensured that allocation concealment was maintained until after patient enrollment despite not blinding the study intervention. We were concerned that pre-enrollment awareness of treatment allocation could lead to selection bias. Importantly, there was no allocation strategy we believed could eliminate cross-over between groups. Selection of fluid type is dictated by multiple factors, including a potential lack of personal equipoise across providers, numerous clinical indications for specified fluid and electrolyte titration, ongoing debate about the utility of acid buffers in acutely ill patients [50, 51], and imperfect awareness of study enrollment. However, a tolerance of some use of non-study fluid after enrollment will mimic real-world conditions where cross-over between fluid types is common. As such, we defined adherence as receipt of ≥75% of total crystalloid administered by type randomized. Therefore, our study is most appropriately construed as the comparative effectiveness between the *predominant*—rather than exclusive—use of balanced/buffered fluids versus 0.9% saline. Even if exclusive use of a single fluid type could be executed under controlled experimental conditions, it is unlikely that such a practice could be generalized to real-world fluid management.

Several potential threats to the validity of our trial exist. First, enrollment of children with suspected septic shock exclusively in the ED will include a wide range of illness severity and fluid exposure. Prior studies have demonstrated that fluid type has a negligible clinical impact when exposure is limited to low volumes [17, 18]. Therefore, we only include children who require > 1 fluid bolus *and* the fluid is directed to treat abnormal perfusion rather than dehydration or hypovolemia. This approach balances early enrollment, when it may not yet be clear how much fluid a patient may require, with enrichment of the study population by children for whom fluid type is most likely to have a clinical impact. In our pilot study using the same enrollment criteria, 42% were admitted to the intensive care unit [25], which represents the population with the highest exposure to study fluid and the greatest risk of achieving the primary outcome. A second threat is the lack of blinding. This decision was a practical one to constrain costs of providing blinded fluid bags across all parts of the hospital and needing additional safeguards if the treating physician was blinded to fluid type. However, we chose objective endpoints unlikely to be influenced by knowledge of study group assignment and will maintain blinding of the lead study statistician. Third, frequent withdrawal after EFIC or deferred consent could limit exposure to the intervention and/or impede outcome assessment. Although studies indicate < 1% withdrawal after EFIC or deferred consent [32, 52] and only 2% withdrew after EFIC in our pilot study [25], we plan to modify the analysis if missing data exceeds expectations. Finally, MAKE30 represents a composite of endpoints that may not be of equivalent value to patients or clinicians.

## Trial status

PRoMPT BOLUS is a pragmatic randomized interventional trial of the effectiveness and safety of fluid resuscitation with balanced/buffered fluids compared to 0.9% saline for children with septic shock as part of usual clinical care. Patient enrollment began on August 25, 2020, and is anticipated for 5 years (clinicalTrials.gov/NCT04102371) under this protocol version 1, dated May 25, 2021.

## Supplementary Information

**Additional file 1.** SPIRIT 2013 Checklist: Recommended Items to Address in a Clinical Trial Protocol and Related Documents

**Additional file 2.** Suggested Unsafe Conditions for Study Enrollment

**Additional file 3.** Data Collection Planned for the PRoMPT BOLUS Pragmatic Clinical Trial

**Additional file 4.** Analysis Plan for Secondary Outcomes

## Data Availability

The trial statisticians (HK, JH), lead data manager (KLH), and study principal investigators (SLW, FB, NK) will have full, unrestricted access to and be responsible for the final dataset. Members of the international steering committee will also have access to the final dataset upon request. All study investigators can request specific elements of the study data, which will require approval by the international steering committee. After the publication of main and secondary results, a public use dataset will be made available through the *Eunice Kennedy Shriver* National Institute of Child Health and Development Data and Specimen Hub (DASH). The study funders will not restrict access to study data.
