# Correction: PRagMatic Pediatric Trial of Balanced vs nOrmaL Saline FlUid in Sepsis: study protocol for the PRoMPT BOLUS randomized interventional trial

**DOI:** 10.1186/s13063-025-09164-3

**Published:** 2025-10-07

**Authors:** Scott L. Weiss, Fran Balamuth, Elliot Long, Graham C. Thompson, Katie L. Hayes, Hannah Katcoff, Marlena Cook, Elena Tsemberis, Christopher P. Hickey, Amanda Williams, Sarah Williamson-Urquhart, Meredith L. Borland, Stuart R. Dalziel, Ben Gelbart, Stephen B. Freedman, Franz E. Babl, Jing Huang, Nathan Kuppermann

**Affiliations:** 1 Department of Anesthesiology and Critical Care, The Children’s Hospital of Philadelphia, Perelman School of Medicine at the University of Pennsylvania, Philadelphia, PA USA; 2 The Children’s Hospital of Philadelphia Pediatric Sepsis Program, Philadelphia, PA USA; 3 Department of Pediatrics, The Children’s Hospital of Philadelphia, Perelman School of Medicine at the University of Pennsylvania, Philadelphia, PA USA; 4 Department of Emergency Medicine, The Royal Children’s Hospital, Parkville, VIC Australia; 5 Departments of Pediatrics and Critical Care, The University of Melbourne, Parkville, VIC Australia; 6 Murdoch Children’s Research Institute, Melbourne, VIC Australia; 7 Departments of Pediatrics and Emergency Medicine, Alberta Children’s Hospital Research Institute, Cumming School of Medicine, University of Calgary, Calgary, AB Canada; 8 Department of Biomedical and Health Informatics, Data Science and Biostatistics Unit, The Children’s Hospital of Philadelphia, Philadelphia, PA USA; 9 Divisions of Emergency Medicine and Pediatrics, Perth Children’s Hospital, School of Medicine at the University of Western Australia, Crawley, Australia; 10 Departments of Surgery and Pediatrics: Child and Youth Health, Starship Children’s Hospital, University of Auckland, Auckland, New Zealand; 11 Australian and New Zealand Intensive Care Society Paediatric Study Group, Camberwell, Australia; 12 Paediatric Intensive Care Unit, Royal Children’s Hospital, Parkville, VIC Australia; 13 Sections of Pediatric Emergency Medicine and Gastroenterology, Departments of Pediatrics and Emergency Medicine, Cumming School of Medicine, University of Calgary, Calgary, AB Canada; 14 Department of Biostatistics, Epidemiology, and Informatics, Perelman School of Medicine at the University of Pennsylvania, Philadelphia, PA USA; 15 Department of Emergency Medicine and Pediatrics, UC Davis School of Medicine and UC Davis Health, Sacramento, CA USA

**Correction: Trials 22, 776 (2021)**

**https://doi.org/10.1186/s13063-021-05717-4**

Following the publication of the original article [1], we were notified that one of the collaborators’ names was misspelled as Koutralis instead of Koutroulis.

**The original article has been corrected.**
