# Supplementary material for: PRagMatic Pediatric Trial of Balanced vs nOrmaL Saline FlUid in Sepsis: study protocol for the PRoMPT BOLUS randomized interventional trial
Source: Trials. 2021 Nov 6;22:776. doi: 10.1186/s13063-021-05717-4 (PMC8572061; doi:10.1186/s13063-021-05717-4)
Supplement: Supplementary file 2 — Additional file 2. Suggested Unsafe Conditions for Study Enrollment [file 13063_2021_5717_MOESM2_ESM.docx]

**Additional file 2: Suggested Unsafe Conditions for Study Enrollment**

| Although final assessment of safe use of either fluid type will be left to the discretion of the treating clinician, the following conditions—if known at time of assessment for eligibility—are suggested reasons for exclusion based on unclear safety: |
| --- |
| - Suspicion for impending brain herniation |
| - Known hyperkalemia (serum or whole blood potassium >6 mEq/L) |
| - Known hypercalcemia (total calcium >12 mg/dL or ionized calcium > 1.35 mmol/L) |
| - Known acute fulminant hepatic failure (alanine aminotransferase >10,000 U/L or total bilirubin >12.0 mg/dL) |
| - Known history of severe hepatic impairment, defined as diagnosis of cirrhosis, “liver failure”, or active listing for liver transplant |
| - Known history of severe kidney disease , defined as current dependency on peritoneal dialysis or hemodialysis |
| - Known metabolic disorder, inborn error of metabolism, or primary mineralocorticoid deficiency (e.g., mitochondrial disorder, urea cycle disorder, amino acidemia, fatty acid oxidation disorder, glycogen storage disorder, congenital adrenal hypoplasia, Addison’s disease) |
