# Supplementary material for: PRagMatic Pediatric Trial of Balanced vs nOrmaL Saline FlUid in Sepsis: study protocol for the PRoMPT BOLUS randomized interventional trial
Source: Trials. 2021 Nov 6;22:776. doi: 10.1186/s13063-021-05717-4 (PMC8572061; doi:10.1186/s13063-021-05717-4)
Supplement: Supplementary file 3 — Additional file 3. Data Collection Planned for the PRoMPT BOLUS Pragmatic Clinical Trial [file 13063_2021_5717_MOESM3_ESM.docx]

**Additional file 3: Data Collection Planned for the PRoMPT BOLUS Pragmatic Clinical Trial**

| **Category** | **Data Elements to be Collected** |
| --- | --- |
| Demographics | Age, race, ethnicity, sex, weight, comorbid conditions |
| Hospital summary | Admission disposition, length of stay, discharge disposition |
| Fluid administration | Crystalloid and colloid fluid volume, timing, and composition, blood product administration, parenteral nutrition |
| Laboratory data | Initial and follow-up blood electrolytes, lactate, total CO_2_ concentration (“bicarbonate”), creatinine, alanine aminotransferase (ALT), baseline creatinine |
| Microbiology | Site of infection, pathogen(s), bacteremia |
| Therapies | Antibiotics (type, timing), vasoactive medications, bicarbonate/acetate (or other acid buffers), corticosteroids, mechanical ventilation, extracorporeal membrane oxygenation |
| Outcomes | Blood creatinine at discharge or 30 days post-enrollment (whichever comes first), inpatient renal replacement therapy, vital status at hospital discharge and 90 days post-enrollment, arterial/venous thromboembolism, cerebral/brainstem herniation |
| Adverse events | Any untoward medical occurrence in a study participant |
