# Supplementary material for: PRagMatic Pediatric Trial of Balanced vs nOrmaL Saline FlUid in Sepsis: study protocol for the PRoMPT BOLUS randomized interventional trial
Source: Trials. 2021 Nov 6;22:776. doi: 10.1186/s13063-021-05717-4 (PMC8572061; doi:10.1186/s13063-021-05717-4)
Supplement: Supplementary file 4 — Additional file 4. Analysis Plan for Secondary Outcomes [file 13063_2021_5717_MOESM4_ESM.docx]

**Additional file 4: Analysis Plan for Secondary Outcomes**

| Secondary outcomes will also be analyzed using the ITT approach. Secondary binary outcomes will be compared using the MH method, stratified by study site. In the case of low outcome counts, the Mantel-Fleiss criterion will be assessed. If the criterion has a value less than 5, the exact version of the MH test will be used and two-sided p-values will be defined as the sum of all probabilities of test statistics with point probabilities that of the observed value or less. We will use the methods of Benjamini and Hochberg to control for the impact of multiple analyses of the secondary outcomes. |
| --- |
| - Persistent kidney dysfunction censored at 30 days will be analyzed as a binary outcome, using the Mantel-Haenszel (MH) method, stratified by study sites. All participants who are known to be alive at 30 days post-intervention will be included. |
| - New inpatient RRT censored at 30 days will be analyzed as a binary outcome, using the MH method, stratified by study sites. All participants who are known to be alive 30 days post-intervention will be included. |
| - Hospital-free days out of 28 (between randomization (day 0) and day 27) will be analyzed as a continuous outcome, using the van Elteren test, stratified by study sites. |
| - All-cause mortality at hospital discharge (censored at 90 days) and all-cause mortality at 90 days will be analyzed as both a binary outcome and a survival outcome. As a binary outcome, these will be analyzed using the MH method, stratified by study site. As a survival outcome, these will be analyzed using the Kaplan Meier estimator by treatment group and study site. Mixed effects Cox regression models will be used to compare the hazard ratio between treatment groups, with random intercepts to account for potential study site effects. Schoenfeld residuals and Kaplan Meier plots will be used to check the proportional hazards assumption. All participants who are known to be alive 30 days post-intervention will be included. |
| - Safety endpoints will be analyzed as binary outcomes, using the MH method, stratified by study sites. |
| - Urine and plasma biomarkers will be analyzed as continuous outcomes, using the van Elteren test, stratified by study site. We will compare absolute values at each study timepoint (day 2 and day 27), as well as changes from the initially measured biomarker values on study day 0. |
